# Impact of Mechanochemical Activation (MChA) on Characteristics and Dye Adsorption Behavior of Sawdust-Based Biocarbons

**DOI:** 10.3390/ma17184458

**Published:** 2024-09-11

**Authors:** Barbara Wawrzaszek, Barbara Charmas, Katarzyna Jedynak, Ewa Skwarek

**Affiliations:** 1 Department of Chromatography, Institute of Chemical Sciences, Faculty of Chemistry, Maria Curie-Sklodowska University, Maria Curie-Sklodowska Sq. 3, 20-031 Lublin, Poland; wawrzaszek.barbara@gmail.com; 2 Institute of Chemistry, Faculty of Natural Sciences, Jan Kochanowski University, Uniwersytecka Str. 7, 25-406 Kielce, Poland; kjedynak@ujk.edu.pl; 3 Department of Radiochemistry and Environmental Chemistry, Institute of Chemical Sciences, Faculty of Chemistry, Maria Curie-Sklodowska University, Maria Curie-Sklodowska Sq. 3, 20-031 Lublin, Poland; ewa.skwarek@mail.umcs.pl

**Keywords:** sawdust, biocarbons, pyrolysis, mechanochemical activation, structural characteristics

## Abstract

The increase in environmental pollution due to the development of industry and human activity has resulted in intensive development of research on the possibility of its purification. A very effective method is the pollutants’ adsorption from the air and water environment. For adsorption to be effective, materials with a specific structure and a well-developed surface decorated with numerous functionalities, e.g., biocarbons (BC), are necessary. An effective method of activating biocarbons is mechanochemical milling, an environmentally friendly procedure. This paper describes the possibility of using mechanochemical activation (MChA) of non-porous biocarbons to develop surface and porosity for their use in processes of pollutant adsorption. BC was characterized based on N_2_ adsorption, thermogravimetry (TGA), SEM/EDS imaging, Fourier (ATR-FTIR) and Raman spectroscopies, as well as titration using the Boehm method and determination of zeta potential. The adsorption capacity of BC for methylene blue (MB) was studied. It was proven that the solvent-free MChA made it possible to obtain microporous biocarbons, causing an intensive increase in the surface area and pore volume and the generation of oxygen functionalities. The biocarbons had predominantly acidic (mainly carboxylic) or basic functionalities and exhibited an amorphous structure. BC proved to be effective in adsorbing MB from aqueous solutions.

## 1. Introduction

Carbon materials (nanotubes, graphene, fullerenes, and porous carbons) attract more and more attention due to their easy accessibility, tunable surface, good conductivity, as well as chemical and physical stability. These characteristics mean they are extensively used in many research areas, including separation [1], catalysis [2], adsorption [3,4,5], and energy storage [6,7,8]. Activated carbons (AC) are relatively low cost and possess desirable properties, and thus they are one of the most commonly applied carbon materials. They can be manufactured taking into account the sustainability issues and the principle of green chemistry, which involve products and processes designs that minimize the use and production of harmful substances. The precursors of this type of material are, among others, natural materials, i.e., biomass, which is produced as a waste material in industry and households. Waste disposal is an important issue, so its use as a precursor of carbon materials is in line with environmental issues.

Environmental pollution caused by the intensive development of industry has prompted scientists to look for effective methods of its purification. Activated carbons obtained from biomass (biocarbons, BC) are an excellent solution to the problems associated with adsorption of air and water impurities. However, effective adsorption on BC depends mainly on the chemical composition (carbon content and heteroatoms, which can constitute additional adsorption centers) and structural characteristics of the carbon skeleton (porous structure and large surface area rich in highly reactive oxygen functionalities). Biocarbons are obtained through the process of pyrolysis. Carbonizates, formed by the thermal conversion of biomass in an oxygen-free atmosphere in the temperature range of 300–800 °C [9], are non-porous. For their use in the adsorption process, it is necessary to increase the surface area and pore volume, as well as introduce surface functionalities. Physical (CO_2_ [10] and H_2_O [11]) or chemical (e.g., H_3_PO_4_ [7,12], KOH [5,13], and ZnCl_2_ [14]) activators can improve the surface structure properties of biocarbon and adsorption capacity, thus being very effective, and they are widely used. However, the use of such activators has numerous disadvantages: (1) chemical activation, which consumes large amounts of harmful activating reagents, such as H_3_PO_4_, KOH, or ZnCl_2_, corrodes equipment, and produces large amounts of wastewater, polluting the environment; (2) chemical activation processes, including breaking, impregnation, carbonization, and leaching of activating reagents, are of low efficiency; (3) physical activation, in most cases by CO_2_ [10] or steam [11], often does not allow for the generation of AC with a very large surface area.

The search for new, “clean” methods of activating carbon materials following the principles of green chemistry resulted in the use of mechanochemistry. This unconventional method converts the mechanical energy generated during the collision of grinding balls into heat, allowing chemical reactions to take place. Mechanochemistry offers numerous advantages over traditional solvent-based procedures, such as simplicity, mild and short process conditions, scaling-up, and essentially no waste products [15]. Performing the reaction without solvents is advantageous because this is an environmentally friendly and low-cost process. Mechanochemistry has been successfully used for porous carbon materials’ synthesis for over twenty years [16,17]. Initially, graphite was used as a starting material. It was found that graphite grinding in ball mills leads to a phase transition from the hexagonal to the turbostratic one in the short term and the amorphous one in the long term [16]. In both turbostratic and amorphous carbons, the components of the graphene layer are distorted, resulting in the formation of a nanoporous structure. In addition, carbon nanoparticles are produced by ball-milling graphite. Aggregation of these particles can also cause nanopores’ formation. In the case of biocarbon activation, a significant advantage is fast and effective shredding, resulting in the formation of new surfaces as well as structural and network defects, which results in intensive surface development and the introduction of new functionalities [18,19]. Ball milling is effective in obtaining ceramic powders [20] and nanomaterials, including those with photocatalytic properties [21,22], in conducting solvent-free organic reactions [23], and in the degradation of pollutants and spent materials [24,25].

However, to the authors’ knowledge, the mechanochemical biocarbon activation is relatively rarely discussed in the literature, but understanding its potential as a “green activation” method opens up enormous possibilities in the area of obtaining and tailoring materials with desired properties, without the use of toxic, aggressive reagents, the release of harmful products, in a shorter time, and with smaller expenses. In this paper, the possibility of using mechanochemical activation of biomass-based biocarbons under conditions free of chemical activators as an alternative, green method of biocarbon activation is investigated. Mixed-wood sawdust was selected as a renewable source of carbon. The biomass sawdust produced during wood processing in households came from deciduous and coniferous trees (1:1 in this study). The material is diverse in terms of elemental composition and inorganic impurities content due to different harvest times, species diversity, storage conditions, and processing. Our research is focused on the examination of the trend of the structure and surface chemistry changes of biocarbons under the influence of mechanochemical activation.

## 2. Materials and Methods

### 2.1. Materials

Sawdust from mixed trees (deciduous/coniferous, ~1/1) collected from the households was used as a carbon precursor. All reagents: hydrochloric acid (HCl—35%), sodium hydroxide (NaOH, purity 98%), sodium carbonate (Na_2_CO_3_, purity 99%), and sodium bicarbonate (NaHCO_3_, purity 99%), purchased from Standard (Poland), were analytical grade. Methylene blue (purity > 82%) was obtained from Chempur (Poland).

### 2.2. Biocarbon Preparation

The sawdust was washed with tap and distilled water for dust removal, and then dried for 24 h at 105 °C. The dried material was ground and fractionated, and the fraction in the range of 1–2 mm was used for the study. The pyrolysis process conducted in a nitrogen atmosphere (flow rate 100 cm^3^/min and heating rate 10 °C/min), to a temperature of 500 °C, was followed by a one-hour isothermal stage. The obtained starting material was designated BC-500. To develop the surface, the materials were mechanochemically activated in a planetary ball mill (Pulverisette 7, Fritsch, Germany). A sample of approximately 2 g of BC-500 biocarbon was placed in a grinding vessel (80 mL) containing 25 silicon nitride beads with a diameter of 10 mm. The speed of grinding was 300 and 800 rpm for 1 and 3 h, changing the direction of mill rotation every 15 min. After the modification, the biocarbons were labeled BC-531, BC-533, BC-581, and BC-583, where the first digit (5) indicates the pyrolysis temperature, the second digit indicates the rotation (3 means 300 rpm and 8 means 800 rpm), and the third digit indicates the grinding time (1 or 3 h). For example, BC-531 is a biocarbon obtained at a temperature of 500 °C, mechanochemically modified at a rotation speed of 300 rpm for 1 h.

### 2.3. Methods

#### 2.3.1. Low-Temperature Adsorption/Desorption Nitrogen

The low-temperature nitrogen adsorption/desorption method (−196 °C; ASAP 2405 analyzer, Micromeritics, Norcross, GA, USA) was used to determine the structural parameters: specific surface area (S_BET_), pore size distribution (PSD), and pore volumes (V_mi_ and V_me_) [26,27,28]. The procedures are included in the Supplementary Material (SM). 

#### 2.3.2. SEM/EDS Analysis

The morphology of the biocarbon surface was examined by means of a Quanta 3D FEG scanning electron microscope (FEI) (FEI, Field Electron and Ion Co., Hillsboro, OR, USA) under the low-vacuum conditions at 5 kV. X-ray spectroscopy with energy dispersion (SED/EDS; acceleration: 20 kV) (EDAX, Mahwah, NJ, USA) was used in the qualitative and quantitative analyses.

#### 2.3.3. Thermogravimetric Analysis

Thermogravimetric analysis was conducted by means of Derivatograph C (Paulik, Paulik and Erdey, MOM, Hungary). The ~30 mg samples were introduced in ceramic crucibles, and the reference substance there was Al_2_O_3_. Some measurements were performed in the air atmosphere (20–1200 °C; heating rate 10 °C/min). The TG and DTA curves were recorded. Moreover, to perform an approximate analysis of the materials, the analyses were also conducted in the atmosphere of N_2_ (20–900 °C; temperature increase: 10 °C/min). The volatile matter content (VM%) was estimated from the TGA data (N_2_; 150–900 °C). The ash content (A%) was found to be a residue after the complete thermal degradation of organic substances in the O_2_ atmosphere. The content of fixed carbon (FC%) was calculated from the relationship: FC% = 100 − (A% + VM%). The parameters were determined referring to the dry matter. The thermostability index was estimated based on the formula: C_thermo_ = %FC/(%FC + %VC) [29].

#### 2.3.4. Raman Spectroscopy

For the degree of the order of the biocarbon structure, the via reflex Raman spectra (Microscope DMLM Leica Research Grade, Reflex, Renishaw, UK) was estimated using an argon laser with a 785 nm wavelength.

#### 2.3.5. FTIR-ATR

The FTIR-ATR spectra were recorded using a 400-FTIR/FT-NIR spectrometer (Perkin-Elmer, Waltham, MA, USA), with the single-diamond reflection and the attenuated total reflection (ATR) endurance cell. The samples were dried and powdered before testing. The spectra were recorded in the range of 4000–650 cm^−1^ with a 4 cm^−1^ resolution.

#### 2.3.6. Potentiometric Titration

The Boehm titration [30] was used for determination of oxygen surface functionalities. Carbon weights ~0.2 g poured with 10 cm^3^ of NaOH, HCl, NaHCO_3_, and Na_2_CO_3_ solutions (0.05 N) were placed in a thermostatic bath (25 °C, 140 rpm, 24 h). After the neutralization process, the concentrations of the solutions were determined by applying the potentiometric titration method (Titrino, Metrohm).

#### 2.3.7. Surface pH

The pH of the biocarbons was examined based on the procedure presented in [31]. Here, ~0.4 g of biocarbon weights was poured with distilled water (20 cm^3^). Then, it was placed in a shaker (25 °C, 140 rpm, 24 h). Next, the pH of the supernatants was measured.

#### 2.3.8. Surface Charge Density Determination

For points of zero charge (pH_pzc_) and surface charge densities, the potentiometric titration method was used [32]. The procedure is included in the Supplementary Material.

#### 2.3.9. Electrophoretic Mobility Measurements

The electrophoretic mobility of the tested biocarbons was estimated using zeta potential (ζ) and the isoelectric point (pH_iep_) [33]. The details are presented in the Supplementary Material.

#### 2.3.10. Adsorption Kinetics

In the adsorption studies, three temperatures (298 K, 308 K, and 315 K) were applied, and methylene blue as an impurity. To discuss the processes taking place, the pseudo-first-order (PFO; Equation S1) and pseudo-second-order (PSO; Equation S2) models, as well as the Webber–Morris intraparticle diffusion model (IPD; Equation S3), were applied [34]. All details of the experimental conditions are included in the Supplementary Material. 

#### 2.3.11. Adsorption Isotherms

The investigation of the adsorption isotherms was performed at 298 K, 308 K, and 315 K. MB concentrations were 100–1400 mg/dm^3^. The adsorption process was described based on the experimental data (Equation S4) using the linear forms of the Langmuir (Equation S5), Freundlich (Equation S6), and Dubinin–Radushkevich (Equation S7) equations [34,35,36,37]. The ε parameter and adsorption energy were calculated (Equations S8 and S9). The thermodynamic functions: free energy (ΔG°), enthalpy (ΔH°), and entropy (ΔS°; Equations S10 and S11), were also determined [38]. All details of the experimental conditions and calculations are included in the Supplementary Material. 

## 3. Results and Discussion

Table 1 presents the results of the elemental EDS analysis of the biocarbons. As biocarbons were obtained from organic waste materials, their elemental composition is closely related to the structure of the starting material. The elemental analysis of EDS showed that the starting biocarbon contained 92.6% carbon and 6.2% oxygen, which is due to the presence of lignin, cellulose, and hemicellulose in the starting material [39]. The data in Table 1 indicate that the mechanochemical modification resulted in a gradual decrease in the carbon content while increasing the oxygen content, but the total carbon and oxygen content of the biocarbons remained stable (~99%). The efficiency of these changes was greater when using a higher mill speed (Table 1). The decrease in the carbon content could result from the degradation of organic structures affected by mechanochemical treatment, causing the volatilization of low-molecular organic compounds, while the increase in the oxygen content was associated with the formation of new surface functionalities. The increase in surface functionalities was also confirmed by the values of atomic ratios (O/C; Table 1), which increased as a result of the mechanochemical modification of biocarbons. The presence of other elements (~1%) resulted from their occurrence in the starting material, and the varying content was due to the heterogeneity of the waste material. In the studied materials, in addition to carbon and oxygen, silicon (0.19–0.35%), potassium (0.11–0.2%), and calcium (0.37–0.55%), as well as Na, Mg, P, and S (at the level of 0.01–0.05%; Table 1), were present.

The increase in the number of surface functional groups was confirmed by neutralizing the functional groups according to the method proposed by Boehm [11,30]. This acid–base titration determines the amount of surface acid or basic oxygen groups occurring on activated carbon, carbon black, graphene, or carbon nanotube surfaces. The method uses NaOH, Na_2_CO_3_, NaHCO_3_, and HCl, and its main idea is the determination of the number of acidic sites, assuming that NaOH neutralizes the carboxyl, lactone, and phenolic groups, Na_2_CO_3_ neutralizes the carboxyl and lactone groups, whereas NaHCO_3_ neutralizes only the carboxyl ones. The number of alkaline groups is determined by HCl titration.

The initial biocarbon was characterized by the acidic and basic groups, with triple the predominance of the latter (Table 2). The mechanochemical modification caused a gradual increase in the number of both types of functional groups [40]. The content of basic functionalities after the initial increase due to the use of MChA remained at a similar level (~0.44 mgR/g). It is worth noting that biocarbons milled at 300 rpm (BC-531 and BC-533) were characterized by a predominance of basic groups, while increasing the rotation rate to 800 rpm (samples BC-581 and BC-583; Table 2) resulted in an intensive increase in acidic groups. The carboxyl groups also appeared to be absent in the starting biocarbon (for BC-533, BC-581, and BC-583). The increase in the number of acidic groups after milling was caused by oxygen chemisorption on the unsaturated carbon atoms at the edges of the graphene layers and on the basal plane defects [40]. The presence of functional groups affected the surface pH, which decreased (from 8.8 to 7.5; Table 2) gradually after the application of MChA (Table 2). In the case of the BC-581 and BC-583 samples, despite the evident predominance of acidic groups, the pH of the materials was ≥7.5. This was probably due to the fact that functional groups of a basic nature are strong bases [31].

**Figure 1:**
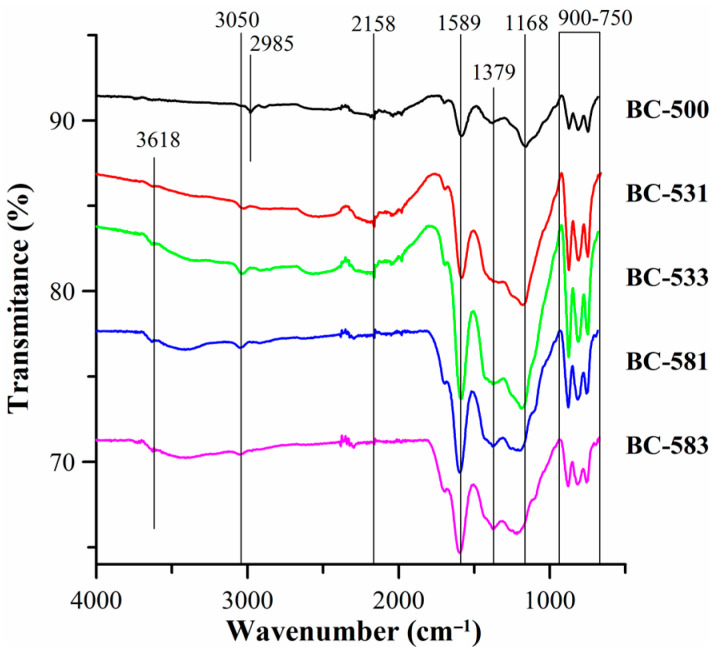
ATR-FTIR spectra of the biocarbons.

The presence of surface functional groups was also confirmed by the FTIR-ATR analysis (Figure 1). The small band occurring at 3618 cm^−1^ for the samples subjected to the mechanochemical modification corresponded to the symmetrical and asymmetrical tensile vibrations of the O-H groups [5]. The band at ~3050 cm^−1^ indicated the tensile vibrations of C-H in the aromatic rings [41]. Occurring at 1589 cm^−1^, the band was associated with the vibrations of C=C or those of the C=O group present in the oxygen functional groups, such as ketones, carboxylic acid groups, and amides [42]. The bands in the range of 1500–1100 cm^−1^ were associated with the presence of C=O carbonyl groups [43]. The bands at 1379 cm^−1^ corresponded to the vibrations of the carboxyl group O=C-O [44], which was confirmed by the Boehm method. The bands occurring in the area of 900–750 cm^−1^ were associated with the C-H deformation vibrations outside the plane of the aromatic rings [45]. For all modified biocarbons, an increase in the intensity of the bands associated with the oxygen-containing groups was observed, which was confirmed by an increase in the content of surface oxygen functional groups for the studied materials.

The purpose of activating biocarbons for their potential applications as materials with good sorption properties is, in addition to activation by the introduction of surface functional groups, the effective development of surface and porosity. The efficiency of surface development depends on the pyrolysis conditions (temperature and time), but it is known that mechanochemical modification makes it possible to obtain biocarbons with a better developed and ordered structure and porosity [46,47]. BC-500 biocarbon, obtained at a temperature of 500 °C, was characterized by a very poorly developed surface area and pore volume, although micropores constitute ~99% of this material (Table 3).

**Figure 2:**
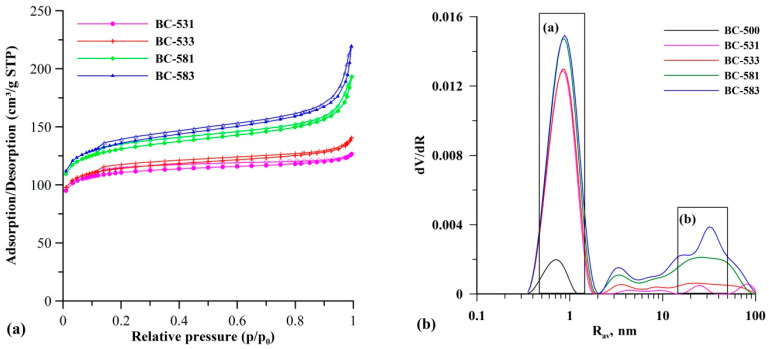
Low-temperature N_2_ adsorption/desorption isotherms (**a**) and pore volume distribution curves (**b**) obtained for the tested biocarbons.

Figure 2 shows the isotherms of low-temperature nitrogen adsorption/desorption (Figure 2a) and pore volume distribution curves (Figure 2b) for the biocarbons subjected to mechanochemical activation. The shape of the isotherms was intermediate between types I and IV after IUPAC. In the low relative pressure range (<0.1), the sorption capacity increased rapidly as p/p_0_ increased. This was due to strong adsorption in the micropores. As the relative pressure increased, the isotherm increased steadily in the medium- and high-pressure areas, and the adsorption and desorption curves began to become inconsistent. This resulted in the formation of a hysteresis loop, indicating the formation of a small number of mesopores [48]. It can be seen that the isotherms were arranged higher and higher (Figure 2a), which suggests that both the extension of the modification time and the increase in the speed of rotation of the mill resulted in better surface development (Figure 2a). The pore volume distribution curves (Figure 2b) indicated an intense increase in the volume of pores with a dominant radius of ~0.8 nm (Figure 2b, inset a) due to the mechanochemical modification and the formation of a mesoporous structure (Figure 2b, inset b), with a simultaneous increase in the value of the mean pore radius (R_av_; Table 3) of the modified biocarbons. The greatest development of the mesoporous structure was observed for the samples milled at 800 rpm (BC-581 and BC-583).

**Figure 3:**
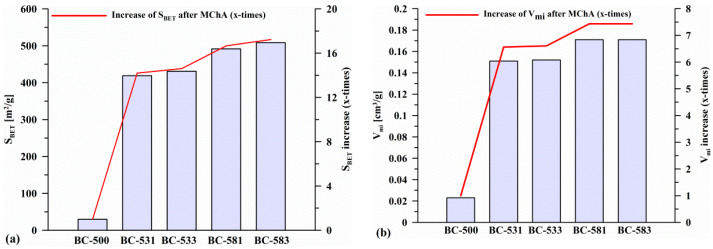
Changes in S_BET_ (**a**) and V_mi_ (**b**) of the initial and mechanochemically activated biocarbons.

The structural parameters determined based on the nitrogen adsorption/desorption isotherms are presented in Table 3. Mechanochemically treated biocarbons had a much larger specific surface area (S_BET_~224.6 to 508.3 m^2^/g, ~14–17 times increase; Figure 3) compared to the initial biocarbon (S_BET_ = 29.5 m^2^/g), which was caused by the breakdown and cracking of biocarbon particles and the formation of a new network of pores, thus increasing the specific surface area of the materials [49,50]. All determined parameters (Table 3) increased due to the grinding process and the increase in the process parameters (time and rotation speed). Since the total surface area was very intensively developed (14–16-fold increase in S_BET_ (Figure 3a) and 6–7-fold increase in V_mi_ (Figure 3b)), a decrease in the share of micropores (%S_mi_ and %V_mi_; Table 3) was observed, with a simultaneous increase in the share of mesopores (%S_me_ and %V_me_; Table 3). This was also confirmed by the hysteresis loops appearing on the isotherms determined for the mechanochemically modified biocarbons. This was due to the degradation of larger biocarbon particles as a result of milling and development of a microporous structure [51]. Increasing the grinding time (from 1 to 3 h, samples BC-531 vs. BC-533 or BC-581 vs. BC-583) did not cause significant changes in the surface. However, significant changes were observed when the rotation speed was increased (from 300 to 800 rpm, e.g., BC-531 and BC-581; Figure 3). 

**Figure 4:**
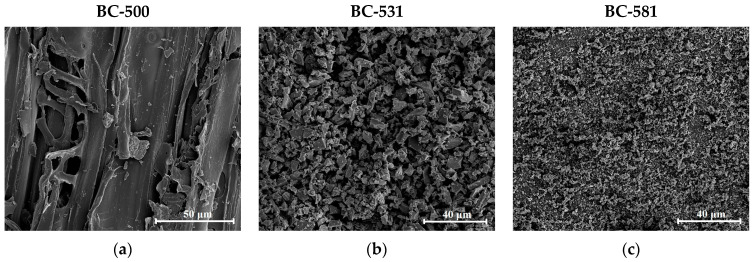

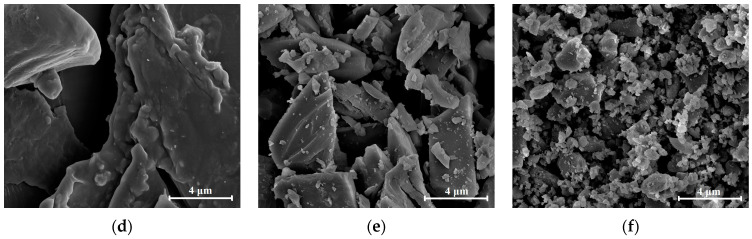
SEM images of the biocarbons: BC-500 (**a**,**d**), BC-531 (**b**,**e**), and BC-581 (**c**,**f**) at 1000× and 10,000× magnification.

The SEM images (Figure 4) of the starting biocarbon (BC-500) and biocarbons treated with MChA (BC-531 and BC-581) clearly showed the changes in the particle morphology due to the mechanochemical modification. The surface of the output biocarbon (Figure 4a) revealed a compact structure, including numerous channels and fissures, indicating that the original precursor structure was preserved in the pyrolysis process [52]. The mechanochemically treated biocarbons took the form of granular, irregular particles in the micrometer range. It can be observed that the increase in the rotation rate caused much more intense changes in morphology, causing the layered structure of the biocarbon to change into ultrafine particles [53].

**Figure 5:**
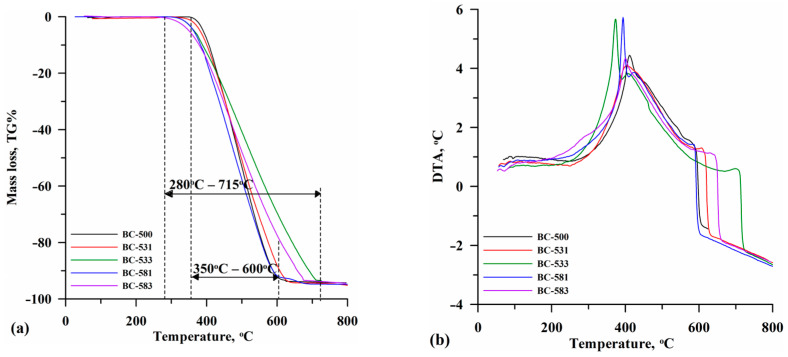
TG% (**a**) and DTA (**b**) results obtained for the tested biocarbons.

Thermal analysis allowed the biocarbon thermal stability assessment (Figure 5). The TG% curves indicated that the thermal degradation occurred in one stage. It started at approximately 350 °C for the baseline BC-500 and approximately 280 °C for the activated biocarbons (Figure 5a). The lower temperature of the onset of thermal decomposition of activated biocarbons could be due to the reduction of biocarbon particles as a result of mechanochemical milling, which improved heat transfer and reduced thermal stability [54]. Faster burning of small biocarbon particles could be the cause of the intense maximum on the DTA curve observed for materials milled for 3 h (BC-533 and BC-583; Figure 5b). The complete decomposition of BC-500 was found at a temperature of approximately 600 °C. The activated biocarbons, especially those ground for 3 h (BC-533 and BC-583), were more thermally resistant, and their decomposition was over at ~700 °C (Figure 5).

The biocarbons were characterized by a small content of inorganic residues (%A; Table 4), which was mainly due to the structure of the carbon precursor. The increase in %A as a result of the application of MChA can be attributed to the degradation of carbon compounds during milling, which is consistent with the results of the elemental analysis presented in Table 1. As follows from the proximate analysis (Table 4), activation increased the volatile carbon (%VC) content with a concomitant decrease in the fixed carbon (%FC) share in the biocarbons, as well as a decrease in the proportion of thermostable fraction (C_thermo_). This was related to the formation of aerobic functional groups in the mechanochemical process, which were easily thermally degraded and reduced the stability of the biocarbons.

**Figure 6:**
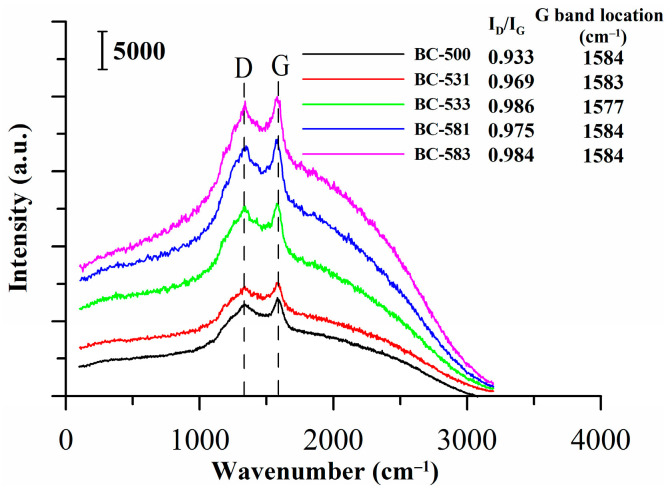
Raman spectra of the biocarbons.

Raman spectroscopy was used to determine the bond type, size of domain, and internal stresses in the amorphous or nanocrystalline carbon materials. Typically, carbons are characterized by wide bands at 1300–1600 cm^−1^. The D-band (~1350 cm^−1^) corresponded to the breathing mode vibrations in the aromatic carbon ring of the nanocrystalline carbon structures. However, the G-band (~1580 cm^−1^) originated from the stretching vibrations of sp^2^ carbon in rings and chain structures. The main parameters determining the type of structure of the carbon material were the position of the G-band and the intensity ratio, I_D_/I_G_ (Figure 6). Based on the relationship between these values, one can state the content of the sp^3^ phase and the type of structure prevailing in the biocarbon [55]. No change in the position of the bands was observed. However, the peak intensities increased slightly. This indicates that the mechanochemical treatment resulted in the formation of biocarbon particles with large structural defects [56]. The calculated I_D_/I_G_ ratios (Figure 6) close to 1 indicate a large number of such defects in the obtained materials. This ratio increased with the grinding time and rotation speed increasing. 

The data presented in Table 2 and Table 3 clearly indicate that the mechanochemically activated carbonaceous materials based on sawdust had a completely different acidic–basic nature of the surface. This indicates their applicability for adsorption purposes. The mechanochemical activation and, particularly, the processing time cause noticeable differences in the textural structure. The pH range from 3 to 11, zeta potential, and surface charge density measurements were typical of this type of material, which neither dissolved under acidic conditions nor precipitated in the tested system under the alkaline conditions. Moreover, the pH_pzc_ values enabled the determination of the pH range in which the surface charge density was negative below pH_pzc_ and positive above pH_pzc_. This was very helpful for characterizing the adsorption process. The ionic strength value in the electrochemical studies was selected in the way enabling the most effective determination of experimental parameters for the electrical double layer, which is consistent with the literature data [57,58].

**Figure 7:**
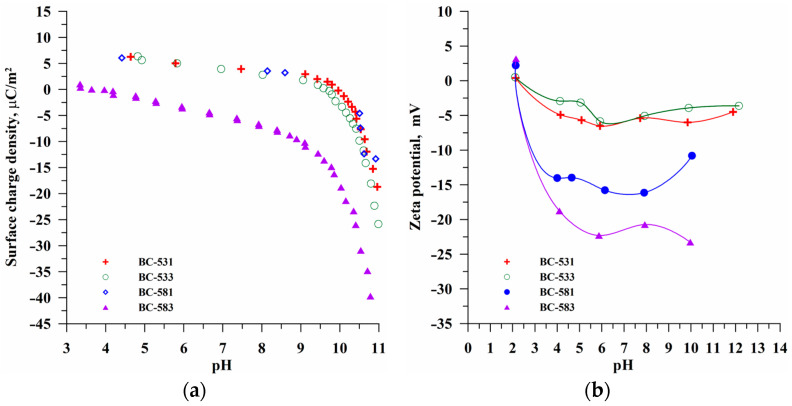
The dependencies of biocarbon particles’ surface charge density (**a**) and zeta potential (**b**) in 0.001 mol/dm^3^ NaCl.

Figure 7 presents the pH impact on the surface charge density (Figure 7a) and zeta potential (Figure 7b) of the tested materials dispersed in the electrolyte. The intersection of the curves with the X-axis enabled the determination of the surface charge density and the zeta potential (ζ) values equal to zero. This enabled us to determine the points of zero charge (pzc) and isoelectric points (iep) of biocarbons. These points, the specific pH values, namely, pH_pzc_ and pH_iep_, are characteristic of each adsorbent–adsorbate system [59]. The pH_pzc_ and pH_iep_ values are presented in Table 5. The pH_pzc_ value for the sample BC-583 was 3.35, which indicates the biocarbon’s acidic nature and the basic nature of the remaining samples.

At the pH values lower than pH_pzc_, the biocarbon surface was positively charged. Thus, the adsorption of negatively charged adsorbates is preferred. At the pH values above pH_pzc_, the solid surface charge had a negative sign, and then the electrostatic attraction with positively charged adsorbates was promoted. The isoelectric points of the tested materials were located at lower pH values and were equal to pH < 2 for all the samples (Figure 7b). A difference between the pH_pzc_ and pH_iep_ values is often observed. This can result from the electrical double layer (EDL) overlapping formed on the opposite adsorbent pores walls, which affects the ionic composition of the diffusion EDL parts [60]. Additionally, in Figure 7b, one can observe that the highest values of the zeta potential were for the BC-583 sample processed at the largest grinder speed and for the longest time. In the pH range of 4 to 11 for this sample, it can be considered colloidal stable.

**Figure 8:**
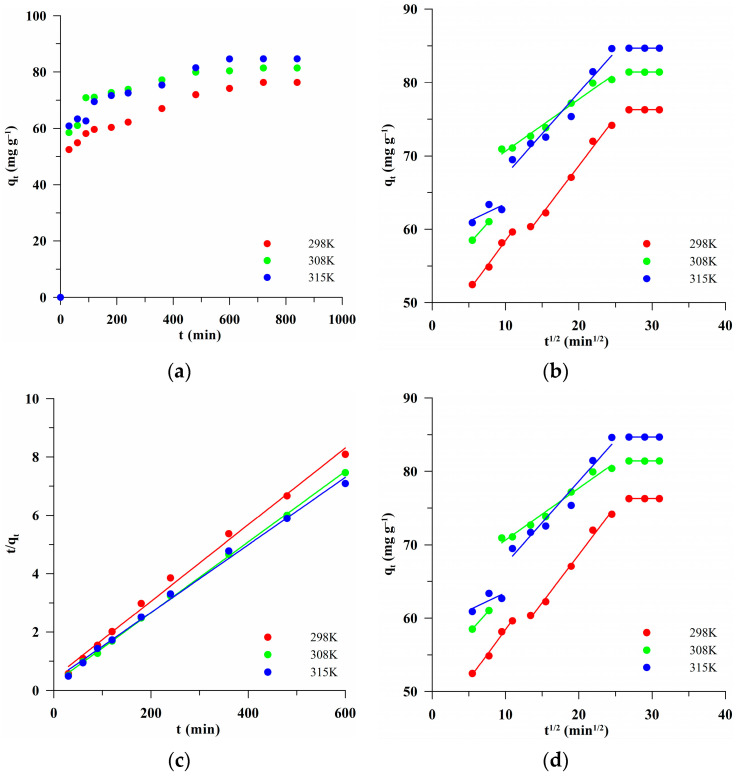
Experimental kinetic data (**a**). Models of pseudo-first-order (**b**) and pseudo-second-order (**c**) kinetics and the intra-particle diffusion model (**d**) at different temperatures for the sample BC-583.

To sum up, considering the electrostatic interactions of the biocarbon and dye, MB (cationic dye) should be strongly adsorbed on the negatively charged carbon surface at pH > pH_pzc_. The pH_pzc_ value for BC-583 was 3.35, which indicates a negatively charged biocarbon surface. Therefore, this carbon was tested for the sorption capacity with regard to MB. It was characterized by the best developed specific surface area (S_BET_ = 508 m^2^/g) and the largest pore volume (V_p_ = 0.34 cm^3^/g). Figure 8 shows the fitting of the experimental data (Figure 8a) to the linear forms of the pseudo-first-order (Figure 8b) and pseudo-second-order (Figure 8c) kinetic equations for the biocarbon. The calculated kinetic parameters and the correlation coefficient (R^2^) are presented in Table 6. The obtained data were much better described by the pseudo-second-order model, as indicated by the higher value of the fit factor (R^2^ > 0.99). Fitting to the PSO model suggested the chemisorption mechanism of the adsorption [61].

The MB adsorption was also characterized by the Webber–Morris intramolecular diffusion model. The course of the relationship q_t_ = f(t^1/2^) (Figure 8d) made it possible to determine the mechanism of adsorption. For all temperatures, multilinear, three-stage relationships were obtained. The first, the fastest one, was related to the transport of dye molecules to the adsorbent surface. Then, the diffusion of dye molecules inside the pores to the adsorbent inner surface took place, as described by the second part of the q_t_ = f(t^1/2^) relationship. The third stage indicated the equilibrium state of the system. The designated IPD parameters are presented in Table 6. The calculated c > 0 parameter suggested that intraparticle diffusion had a significant impact on adsorption but was not a speed-determining step [62].

**Figure 9:**
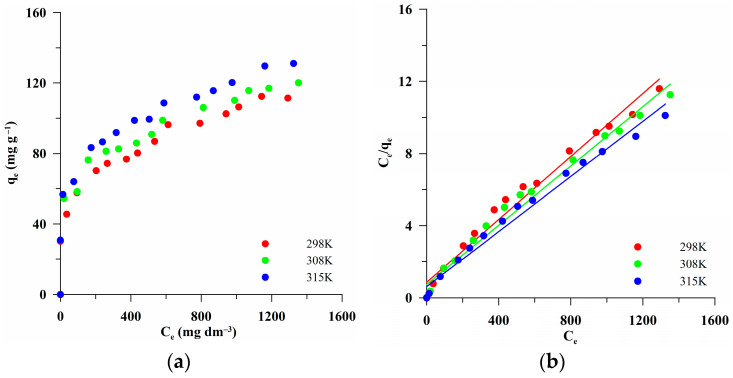

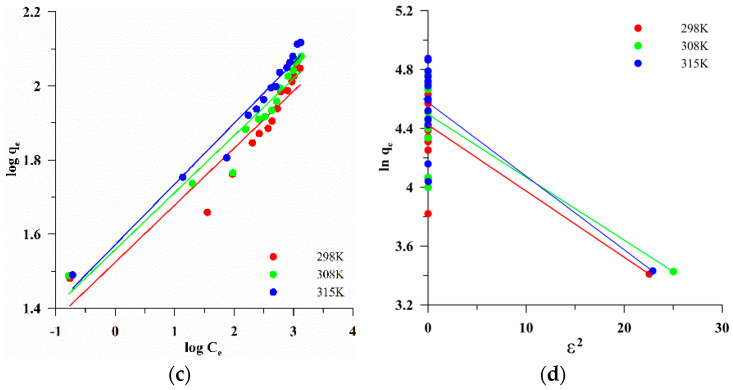
Experimental MB adsorption isotherms (**a**) and linear fittings of isotherms according to the Langmuir (**b**), Freundlich (**c**), and Dubinin–Radushkevich (**d**) models for the sample BC-583.

To describe the adsorption process, experimental adsorption data (Figure 9a) were fitted to the linear models of adsorption isotherms: the Langmuir (Figure 9b), Freundlich (Figure 9c), and Dubinin–Radushkevich (Figure 9d) ones. The initial data and the standard deviations are presented in Table S1 in the Supplementary Material (SM). With the temperature increases, an increase in the value of q_e,exp_ was observed. This indicated that in this case, adsorption was an endothermic process. The maximum sorption capacity (130.98 mg/g) occurred at a temperature of 315 K. This could be due to the greater mobility of the dye particles at a higher temperature, which facilitates diffusion into the inner layer of the adsorbent. The parameters of the equations determined based on linear adjustments of the Langmuir, Freundlich, and Dubinin–Radushkevich isotherms are presented in Table 7. High values of the R^2^ > 0.97 suggested that the adsorption process was better characterized by the Langmuir equation. Determination of the adsorption energy (E; Table 7) based on the Dubinin–Radushkevich model was intended to find out whether the MB adsorption process on the tested adsorbent proceeded as physical or chemical adsorption. The E values smaller than 8 kJ/mol indicate physical adsorption, while those in the range of 8–16 kJ/mol point to chemical adsorption [37]. The obtained E values changed with the increasing temperature, being 3.33, 3.41, and 3.26 kJ/mol (Table 7). However, the obtained results did not indicate a type of adsorption. The small R^2^ coefficient (0.52–0.58) and significantly lower values (q_m,cal_) compared to the experimental data (q_m,exp_; Table 7) indicated that this model is not suitable for the interpretation of the tested process.

Thermodynamics of the adsorption process is another key aspect that makes understanding the adsorption nature possible. The thermodynamic parameters of the adsorption process (the free enthalpy (∆G°), enthalpy (∆H°), and entropy (∆S°)) were calculated (Table 8). 

The values of ∆G° (<0) indicated that the adsorption was a spontaneous process [63], and a decrease in ∆G° with the increasing temperature suggested an increase in the process spontaneity. This could be related to the increase in the energy of the adsorbate molecules, which affected the adsorption process. The positive value ∆H° testified to the endothermic nature of the process [64]. As follows from the positive value ΔS°, completion of the dye adsorption proceeded according to the degree of disorder, which increased within the solid/liquid interface. This could be the result of adsorbed water molecules’ displacement per adsorbed MB molecule, leading to an increase in the system freedom degree, treated as a whole [65]. 

## 4. Conclusions

It was proven that the effectiveness and efficiency of surface development and porosity in the process of mechanochemical solvent-free activation of biocarbons depend on the applied conditions (time and speed of mill rotation). Materials with an amorphous structure, microporous structure, well-developed surface (419–508 m^2^/g), and pore volume (0.196–0.34 cm^3^/g), decorated with surface functionalities (mainly acidic), were obtained. The most effective structural changes were observed during the 3 h activation at 800 rpm. As follows from the electrokinetic studies, biocarbons can adsorb pollutants effectively. It was proven that the MB adsorption proceeded according to the PSO model as well as the Langmuir theory, and intramolecular diffusion had a significant impact on the desorption speed; however, it was not a limiting process. 

## Figures and Tables

**Table 1:** The elemental composition (%w/w) of the selected biocarbons.

Biocarbon	C (%)	O (%)	Na (%)	Mg (%)	Al (%)	Si (%)	P (%)	S (%)	K (%)	Ca (%)	O/C *
BC-500	92.62	6.22	0.03	0.08	0.05	0.19	0.04	0.02	0.20	0.55	0.05
BC-531	90.48	8.67	0.03	0.06	0.05	0.18	0.03	0.02	0.11	0.37	0.07
BC-581	88.14	10.8	0.05	0.08	0.08	0.35	0.01	0.01	0.11	0.37	0.9

O/C *—The atomic ratio based on the EDS analysis.

**Table 2:** The surface functionalities and supernatant pH values of biocarbons.

Biocarbon	Acidic Surface Groups *			Σ_acidic_ *	Σ_basic_ *	Σ *	pH
	Carboxyl *	Phenolic *	Lactone *				
BC-500	-	0.0769	0.0274	0.1043	0.3128	0.4171	7.2
BC-531	-	0.1251	0.0410	0.1661	0.4285	0.5946	8.8
BC-533	0.0474	0.2089	0.0589	0.3152	0.4316	0.7468	8.5
BC-581	0.0248	0.3499	0.1298	0.5080	0.3844	0.8836	8.0
BC-583	0.1214	0.5156	0.2162	0.8533	0.4575	1.3108	7.5

* (mgR/g); Σ—The total surface oxygen groups.

**Table 3:** The structural characteristics of the obtained biocarbons.

Carbon	S_BET_	S_mi_	%S_mi_	S_me_	%S_me_	V_p_	V_mi_	%V_mi_	V_me_	%V_me_	Δw	R_av_
BC-500	29.5	29.2	99.0	0.2	0.7	0.023	0.023	98.7	0.001	1.3	0.515	0.68
BC-531	419.0	370.2	88.4	48.7	11.6	0.196	0.151	77.2	0.039	20.1	0.247	2.95
BC-533	430.9	372.0	86.3	58.5	13.6	0.217	0.152	69.8	0.055	25.4	0.252	3.87
BC-581	491.7	413.8	84.2	76.5	15.6	0.299	0.171	57.1	0.095	31.9	0.242	7.15
BC-583	508.3	414.5	81.5	91.6	18.0	0.340	0.171	50.4	0.118	34.6	0.249	9.18

Here, S_BET_—the specific surface area (m^2^/g); S_mi_—the micropore surface (m^2^/g); %S_mi_—the share of micropore surface (%); S_me_—the mesopore surface (m^2^/g); %S_me_—the share of mesopore surface (%); V_p_—the pore volume determined as the sum of the micro-, meso-, and macro-pore volumes (cm^3^/g); V_mi_—the micropore volume (cm^3^/g); %V_mi_—the share of micropore volume (%); V_me_—the mesopore volume (cm^3^/g); R_av_—the average pore radius (nm); Δw—the deviation from the assumed slit-shaped pore model.

**Table 4:** The proximate analysis (%A, %VC, and %FC) of mechanochemically activated biocarbons, as well as the thermostability index determined using TGA.

Biocarbon	%A	%VC	%FC	C_thermo_
BC-500	5.6	13.0	81.4	0.862
BC-531	2.1	19.8	78.1	0.796
BC-533	3.5	17.0	79.5	0.828
BC-581	5.1	18.0	76.9	0.818
BC-583	8.3	21.0	70.7	0.778

**Table 5:** The parameters of the electrical double layer.

Sample	pH_pzc_	pH_iep_
BC-531	9.94	<2
BC-533	9.65	<2
BC-581	8.62	<2
BC-583	3.35	<2

**Table 6:** The kinetics parameters of PFO, PSO, and IPD models for the adsorption of methylene blue on BC-583.

Temp. (K)	PFO Model		PSO Model		IPD Model					
	k_1_	R^2^	k_2_	R^2^	k_id1_	c_1_	R^2^	k_id2_	c_2_	R^2^
298	0.004	0.9564	0.0004	0.9947	1.36	44.80	0.9853	1.32	42.36	0.9912
308	0.0053	0.9675	0.0006	0.9993	1.17	52.39	1.0000	0.71	63.57	0.9780
315	0.0086	0.7435	0.0004	0.9953	0.48	58.71	0.5594	1.13	56.15	0.9538

Here, k_1_—the pseudo-first-order rate constant (min^−1^); k_2_—the pseudo-second-order rate constant (g mg^−1^ min^−1^); R^2^—the correlation factor; k_id_—the intraparticle diffusion rate constant (mg g^−1^ min^1/2^); c—the boundary layer thickness (mg g^−1^).

**Table 7:** The parameters of the Langmuir, Freundlich, and Dubinin–Radushkevich adsorption isotherm models.

Model	Parameter	Temperature (K)		
		298	308	315
Langmuir	q_m,exp_	112.10	120.08	130.98
	q_m,cal_	114.94	121.95	131.58
	K_L_	0.0102	0.0112	0.0123
	R^2^	0.9788	0.9824	0.9826
Freundlich	K_f_	33.6589	36.1576	37.2563
	n	6.618	6.506	6.105
	R^2^	0.9027	0.9410	0.9697
Dubinin–Radushkevich	q_m,cal_	83.40	89.59	89.60
	β	0.045	0.043	0.047
	E	3.33	3.41	3.26
	R^2^	0.5230	0.5772	0.5777

Here, q_m,exp_—the amount of adsorbed substance per 1 g of adsorbent in the equilibrium state (mg g^−1^); q_m,cal_—the maximum amount of adsorbed substance (mg g^−1^); K_L_—the Langmuir adsorption equilibrium constant (dm^−3^ mg^−1^); K_f_—the Freundlich constant, which indicates the adsorption capacity (mg^1–1/n^ L^1/n^ g^−1^); n—the empirical constant describing the adsorbent surface heterogeneity; β—the constant associated with the sorption energy (mol^2^/kJ^2^); ε—the Polanyi potential; R—the gas constant (kJ/mol K); T—the absolute temperature (K); E—the adsorption energy (kJ/mol).

**Table 8:** The thermodynamic parameters.

Temperature (K)	∆G°	ΔH°	ΔS°
298	−20.27	15.01	134.13
308	−20.49		
315	−20.74		

Here, ∆G°—the free energy (kJ/mol); ∆H°—the enthalpy (kJ/mol); ∆S°—the entropy (J/mol*K).

## Data Availability

Data are contained within the article and Supplementary Materials.

## Supplementary Materials

The following supporting information can be downloaded at: https://www.mdpi.com/article/10.3390/ma17184458/s1, Methods; Table S1: Experimental results of adsorption isotherms for the BC-583 material.
