# Supplementary material for: Impact of Mechanochemical Activation (MChA) on Characteristics and Dye Adsorption Behavior of Sawdust-Based Biocarbons
Source: Materials (Basel). 2024 Sep 11;17(18):4458. doi: 10.3390/ma17184458 (PMC11433557; doi:10.3390/ma17184458)
Supplement: Supplementary file 1 [file materials-17-04458-s001.zip › materials-3177692-supplementary.pdf]

Supporting Information

Impact of Mechanochemical Activation (MChA) on Characteristics and Dye Adsorption Behavior of Sawdust-Based Biocarbons

Barbara Wawrzaszek [1], Barbara Charmas [1,*], Katarzyna Jedynak [2] and Ewa Skwarek [3]

[1] Department of Chromatography, Institute of Chemical Sciences, Faculty of Chemistry, Maria Curie-Sklodowska University, Maria Curie-Sklodowska Sq. 3, 20-031 Lublin, Poland; wawrzaszek.barbara@gmail.com

[2] Institute of Chemistry, Faculty of Natural Sciences, Jan Kochanowski University, Uniwersytecka Str. 7, 25-406 Kielce, Poland; kjedynak@ujk.edu.pl

[3] Department of Radiochemistry and Environmental Chemistry, Institute of Chemical Sciences, Faculty of Chemistry, Maria Curie-Sklodowska University, Maria Curie-Sklodowska Sq. 3, 20-031 Lublin, Poland; ewa.skwarek@mail.umcs.pl

* Correspondence: barbara.charmas@mail.umcs.pl

Methods:

Low-Temperature Adsorption/Desorption Nitrogen

The low-temperature nitrogen adsorption/desorption method (−196 °C, ASAP 2405 analyzer, Micromeritics, Norcross GA, USA) was used to determine structural parameters. Based on the resulting data, the specific surface area (S_{BET}) was determined [1]. The data of nitrogen desorption were applied for determination of the pore size distributions (PSD), differential $f_V(R)$~dV_p/dR based on the model of complex pores with the slit-shaped ones predominance. There was applied the self-consistent regularization (SCR) procedure with the non-negativity condition ($f(R_p) > 0$ at any pore radius R) and the regularization parameter $\alpha = 0.01$ calculations. To calculate the contributions of micropores (V_{mi} and S_{mi} at $R \leq 1$ nm) and mesopores (V_{me} and S_{me} at 1 nm < R < 25 nm) to the total pore volume as well as the specific surface area the $f_V(R)$ functions were used. The total pore volume V_p was found as a sum of V_{mi}, V_{me} and V_{ma} values. The formula: ($S_{BET}/(S_{slit}$-1)) [2,3] was applied for the calculation of the Δw which is the deviation from the assumed pore model.

Surface Charge Density Determination

The method of potentiometric titration was used to determine of both surface charge densities of the activated biocarbons and their points of zero charge (pH_{pzc}). There were prepared suspensions of 50 cm^3 of 0.001 mol/dm^3 NaCl containing 0.095g of BC-531, 0.093g of BC-533, 0.081g of BC-581 or 0.079g of the BC-583 activated biocarbon (this provided 40 m^2 of the tested adsorbent in the measuring cup). The measurements took place at 25 °C in the 3–11 pH range. After titrant adding (0.1 mol/dm^3 NaOH portions) changes in the suspension pH were monitored (pHM 240 pH meter, Radiometer, Poland) equipped with glass and calomel electrodes (Beckman Instruments, Brea, CA, USA). The changes related to the surface charge density value as a solution pH function were calculated based on the "Titr_v3" procedure, discussed in detail in [4].

Electrophoretic Mobility Measurements

The electrophoretic mobility of particles of activated carbon by determination of the zeta potential (ζ) and isoelectric point (pH_{iep}) was conducted employing a Zetasizer Nano ZS (Malvern, United Kingdom). For ζ potential calculation, the Smoluchowski equation [5] was applied. 200 cm^3 suspensions containing the electrolyte (C = 200 ppm) and 0.03g of the activated biocarbon were prepared. Each system was dispersed under ultrasound action (Misonix, Farmingdale, NY, USA) and divided into 7 parts. The measurements were made at 25 °C in the pH range from 3 to 10. The appropriate pH of the suspension was established using the 0.1 mol/dm^3 HCl or NaOH solution. The measurements were

made using Φ360 pH meter (Beckman, CA, USA). Thus the dependencies of the zeta potential as a function of the solution pH were obtained.

Adsorption kinetics

In the adsorption studies there were applied three temperatures (298 K, 308 K and 315 K), and methylene blue as an impurity. The ~0.02g biocarbons weights were poured with a methylene blue solution (C_0 = 500 mg/dm^3, V = 10 ml) and then put into a shaker (140 rpm, 0.5-12 h). Next the samples were filtered (MicroPore, 0.45 μm) and the dye concentration was spectrophotometrically determined ("Helios Gamma", Spectro-Lab, Poland) at a 664 nm wavelength. The experiments were repeated three times. To discuss the processes taking place there were applied the pseudo-first-order (PFO, Eq. S1) and pseudo-second-order (PSO, Eq. S2) models as well as the Webber-Morris intraparticle diffusion model (IPD, Eq. S3) [6].

$$\ln(q_e - q_t) = \ln q_e - k_1 t \quad \text{(Eq. S1)}$$

$$t/q_t = 1/(k_2 q_e^2) + t/q_e \quad \text{(Eq. S2)}$$

$$q_t = k_{id} t^{1/2} + C \quad \text{(Eq. S3)}$$

where: q_t — the amount of adsorbed substance per 1 gram of adsorbent after time t [mg g^{-1}]; q_e — the amount of adsorbed dye per 1 gram of adsorbent at equilibrium [mg g^{-1}]; k_1 - the reaction rate constant [min^{-1}]; k_2 — the reaction rate constant [g mg^{-1} min^{-1}]; k_{id} — the intraparticle diffusion rate constant [mg g^{-1} min$^{1/2}$]; C — the boundary layer thickness [mg g^{-1}]; t — the adsorption time [min].

Adsorption Isotherms

MB solutions with the concentrations of 100–1400 mg/dm^3 were used to determine the adsorption isotherms. ~0.02 g of biocarbon was added into the 100 ml Erlenmayer flasks and poured with 10 ml of dye solution. Then it was placed in a shaker (140 rpm, 12 h). The measurements were carried out at three temperatures: 298 K, 308 K and 315 K. The experiments were repeated three times. The adsorption capacity of the studied biocarbon was calculated from the equation (Eq. S4):

$$q_e = ((C_0 - C_e) \cdot V)/m \quad \text{(Eq. S4)}$$

where: q_e — the amount of MB adsorbed dye per 1 g of biocarbon at equilibrium state [mg g^{-1}]; C_0 — the concentration of the MB initial solution [mg dm^{-3}]; C_e — the equilibrium concentration of the MB [mg dm^{-3}]; V— the MB solution volume [dm^3]; m — the sample mass [g].

The adsorption process was determined by the experimental data fitting to the linear forms of the Langmuir (Eq. S5), Freundlich (Eq. S6) [7,8] and Dubinin–Radushkevich (Eq. S7) [9] equations:

$$C_e/q_e = 1/q_m \times C_e + 1/(q_m \times K_L) \quad \text{(Eq. S5)}$$

$$\log q_e = \log K_F + 1/n \log C_e \quad \text{(Eq. S6)}$$

$$\ln q_e = \ln q_m - \beta \varepsilon^2 \quad \text{(Eq. S7)}$$

The parameter ε was defined from the equation:

$$\varepsilon = RT \ln(1 + 1/C_e) \quad \text{(Eq. S8)}$$

The adsorption energy (E), expressed as the free energy transfer of 1 mol of solute from the infinity of the adsorbent surface [10] was determined based on the equation:

$$E = 1/\sqrt{2\beta} \quad \text{(Eq. S9)}$$

where: q_e — the amount of MB adsorbed per 1 gram of adsorbent in the equilibrium state [mg g^{-1}]; q_m — the maximum amount of adsorbed substance [mg g^{-1}]; C_e — the equilibrium concentration of the dye solution [mg dm^{-3}]; K_L — the Langmuir adsorption equilibrium constant [dm^3 mg^{-1}]; K_F — the Freundlich constant which indicates the adsorption capacity [mg$^{1-1/n}$ (dm^3)$^{1/n}$ g^{-1}]; n - the empirical constant describing the adsorbent surface

heterogeneity; β — the constant associated with the sorption energy [mol^2/kJ^2]; ε — the Polanyi potential; R — the gas constant [kJ/mol K]; T — the absolute temperature [K]; E — the adsorption energy [kJ/mol].

The adsorption process was described also from the determined thermodynamic functions such as free energy (ΔG°), enthalpy (ΔH°) and entropy (ΔS°) [11].

$$\Delta G° = -RT\ln K_d \qquad \text{(Eq. S10)}$$

$$\ln K_d = -(\Delta H°/R * 1/T) + \Delta S°/R \qquad \text{(Eq. S11)}$$

where: ΔG° — the free energy (kJ/mol); ΔH° — the enthalpy (kJ/mol), ΔS° — the entropy (J/mol*K); R — the universal gas constant (8.314 J/mol*K); T — the absolute temperature (K); K_d — the equilibrium constant equal to q_e/C_e. Drawing the van't Hoff plot of $\ln K_d$ versus 1/T makes determination of the ΔH° and ΔS° values possible from the slope and intercept.

Table S1. Experimental results of adsorption isotherms for the BC-583 material.

C_0 (mg/dm³)	298K $C_e \pm \sigma$ (mg/dm³)	$q_e \pm \sigma$ (mg/g)	308K $C_e \pm \sigma$ (mg/dm³)	$q_e \pm \sigma$ (mg/g)	315K $C_e \pm \sigma$ (mg/dm³)	$q_e \pm \sigma$ (mg/g)
100	0.17 ± 0.0303	30.26 ± 0.0502	0.17 ± 0.0073	30.76 ± 0.0036	0.19 ± 0.0012	30.90 ± 0.0119
200	35.57 ± 4.8492	45.60 ± 1.6339	19.84 ± 0.8476	54.53 ± 0.4196	13.75 ± 1.5768	56.72 ± 1.4490
300	93.94 ± 5.1746	57.80 ± 1.7328	95.24 ± 5.2689	58.28 ± 2.4963	75.42 ± 7.2420	64.00 ± 4.6667
400	203.25 ± 0.0803	70.22 ± 0.0384	157.65 ± 8.3371	76.32 ± 4.0276	174.67 ± 11.2154	83.35 ± 6.7279
500	265.70 ± 8.0775	74.36 ± 4.8983	259.38 ± 12.5792	81.38 ± 5.4569	239.11 ± 17.1690	86.63 ± 5.2182
600	374.93 ± 20.8933	76.80 ± 7.2626	329.81 ± 4.2375	82.64 ± 3.8882	316.65 ± 9.4664	91.88 ± 4.6880
700	437.63 ± 20.4648	80.35 ± 10.3841	431.52 ± 8.6295	85.95 ± 3.8436	420.62 ± 7.4458	98.81 ± 1.6447
800	535.09 ± 10.2484	86.89 ± 4.0060	518.84 ± 18.8277	90.89 ± 5.3425	504.80 ± 16.0104	99.52 ± 5.8712
900	611.35 ± 4.0530	96.38 ± 1.9141	581.25 ± 9.7104	98.91 ± 2.6079	587.78 ± 7.9382	101.66 ± 3.7421
1000	792.04 ± 10.0694	97.21 ± 2.5055	811.57 ± 4.0421	106.11 ± 2.1849	833.30 ± 11.9901	103.00 ± 0.4171
1100	940.45 ±9.0580	102.57 ± 3.8509	989.75 ± 14.5624	110.16 ± 8.4387	967.90 ± 11.7690	101.66 ± 2.390
1200	1012.45 ±2.3240	106.39 ± 0.9296	1069.60 ± 14.2617	115.69 ± 3.5160	1075.26 ± 8.4022	95.22 ± 3.6054
1300	1142.73 ± 6.7137	112.40 ± 3.3401	1184.39 ± 7.4634	117.00 ± 3.7131	1160.90 ± 8.2837	109.61 ± 4.7023
1400	1291.63 ± 9.2929	111.43 ± 4.4678	1351.64 ± 16.2162	120.08 ± 3.8126	1324.50 ± 11.6461	130.98 ± 3.7424

References

1. Gregg, S.J.; Sing, K.S.W. Adsorption, Surface Area and Porosity, 2nd ed.; Academic Press: London, 1982., doi:10.1002/bbpc.19820861019.
2. Gun'ko, V.M.; Mikhalovsky, S.V. Evaluation of Slitlike Porosity of Carbon Adsorbents. *Carbon* **2004**, *42*, 843–849, doi:10.1016/j.carbon.2004.01.059.
3. Gun'ko, V.M.; Do, D.D. Characterisation of Pore Structure of Carbon Adsorbents Using Regularisation Procedure. *Colloids and Surfaces A: Physicochemical and Engineering Aspects* **2001**, *193*, 71–83, doi:10.1016/S0927-7757(01)00685-9.
4. Janusz, W. Adsorption of Sodium and Chloride Ions at the Rutile/Electrolyte Interface — Parameters of the Electric Double Layer. *Materials Chemistry and Physics* **1989**, 24, 39–50, doi:10.1016/0254-0584(89)90044-8.
5. Paientko, V.; Oranska, O.I.; Gun'ko, V.M.; Skwarek, E. Selected Textural and Electrochemical Properties of Nanocomposite Fillers Based on the Mixture of Rose Clay/Hydroxyapatite/Nanosilica for Cosmetic Applications. *Molecules* **2023**, *28*, 4820, doi:10.3390/molecules28124820.
6. Benjelloun, M.; Miyah, Y.; Akdemir Evrendilek, G.; Zerrouq, F.; Lairini, S. Recent Advances in Adsorption Kinetic Models: Their Application to Dye Types. *Arabian Journal of Chemistry* **2021**, *14*, 103031, doi:10.1016/j.arabjc.2021.103031.

7. Benjelloun, M.; Miyah, Y.; Akdemir Evrendilek, G.; Zerrouq, F.; Lairini, S. Recent Advances in Adsorption Kinetic Models: Their Application to Dye Types. *Arabian Journal of Chemistry* **2021**, *14*, 103031, doi:10.1016/j.arabjc.2021.103031.
8. Chen, S.; Qin, C.; Wang, T.; Chen, F.; Li, X.; Hou, H.; Zhou, M. Study on the Adsorption of Dyestuffs with Different Properties by Sludge-Rice Husk Biochar: Adsorption Capacity, Isotherm, Kinetic, Thermodynamics and Mechanism. *Journal of Molecular Liquids* **2019**, *285*, 62–74, doi:10.1016/j.molliq.2019.04.035.
9. Bazan-Wozniak, A.; Cielecka-Piontek, J.; Nosal-Wiercińska, A.; Pietrzak, R. Adsorption of Organic Compounds on Adsorbents Obtained with the Use of Microwave Heating. *Materials* **2022**, *15*, 5664, doi:10.3390/ma15165664.
10. Kurdziel, K.; Raczyńska-Żak, M.; Dąbek, L. Equilibrium and Kinetic Studies on the Process of Removing Chromium(VI) from Solutions Using HDTMA-Modified Halloysite. *Desalination and Water Treatment* **2019**, *137*, 88–100, doi:10.5004/dwt.2019.23164.
11. Ebisike, K.; Elvis Okoronkwo, A.; Kanayo Alaneme, K.; Jeremiah Akinribide, O. Thermodynamic Study of the Adsorption of Cd^{2+} and Ni^{2+} onto Chitosan – Silica Hybrid Aerogel from Aqueous Solution. *Results in Chemistry* **2023**, *5*, 100730, doi:10.1016/j.rechem.2022.100730.
